# Stellate Ganglion Block for Anosmia and Dysgeusia Due to Long COVID

**DOI:** 10.7759/cureus.27779

**Published:** 2022-08-08

**Authors:** Gaurav Chauhan, Aman Upadhyay, Suchit Khanduja, Trent Emerick

**Affiliations:** 1 Anesthesiology and Perioperative Medicine, University of Pittsburgh Medical Center Presbyterian, Pittsburgh, USA; 2 Pain Management, Pain Clinic of Michigan, Rochester Hills, USA; 3 Anesthesiology, Beaumont Hospital, Southfield, USA; 4 Pain Medicine, University of Pittsburgh Medical Center, Pittsburgh, USA

**Keywords:** dysautonomia, dysgeusia, anosmia, long-covid, covid-19

## Abstract

Anosmia and parosmia refer to the loss or dysfunction of smell, respectively. Dysgeusia refers to taste disturbance. The coronavirus disease 2019 (COVID-19) pandemic and the subsequent phenomenon of Long COVID syndrome have been associated with an increased incidence of anosmia and dysgeusia. Smell and taste disturbances associated with COVID-19 are usually self-limiting but can persist for longer periods in some cases. Imbalances of the autonomic nervous system, especially dysregulation of the sympathetic system, are implicated in the persistence of anosmia and dysgeusia post-COVID-19 infection. Stellate ganglion block (SGB) can diminish the increased sympathetic activity and potentially resolve anosmia and dysgeusia occurring due to Long COVID. The authors report the successful resolution of persistent anosmia and dysgeusia due to Long COVID in a female patient after she underwent SGB.

## Introduction

Anosmia and parosmia are defined as the loss or dysfunction of smell, respectively. Dysgeusia refers to taste disturbance. There is an increased prevalence of anomia/parosmia in patients who have clinically recovered from coronavirus disease 2019 (COVID-19) infection. According to the current literature, the incidence rate of olfactory dysfunction in COVID-19 patients varies from 33.9 to 68%, with a female predominance [1]. Anosmia and dysgeusia could be self-limiting over a two- or three-week period post-COVID-19 infection but can persist for longer. Despite a high recovery rate, multiple studies have reported that up to 7% of the patients still experience smell and taste disturbances more than 12 months after the onset [2,3]. Anosmia and dysgeusia might coexist with other symptoms such as fatigue, orthostatic hypotension, shortness of breath, insomnia, anxiety, and depression. These constellations of symptoms may persist chronically and are termed "Long COVID", or formally, post-acute sequelae of SARS-CoV-2 (PASC) infection [3]. The incidence of PASC is 30% in symptomatic and 5% in asymptomatic patients with COVID-19 infection [4]. Compared to influenza, COVID-19 is reported to be associated with a higher prevalence of anosmia (53% vs. 17%) [5]. PASC patients with or without hospitalization have often reported anosmia and dysgeusia as one of the predominant and persisting symptoms [4].

Smell and taste disturbances associated with COVID-19 infection have generated a lot of interesting etiological hypotheses. One such theory implicates autonomic dysregulation or dysautonomia as the underlying mechanism behind anosmia [6]. In the case of Long COVID, dysautonomia could be induced by the autonomic nervous system's (ANS) response or maladaptation to pro-inflammatory cytokines leading to excessive sympathetic nervous system activity [4,6,7]. Sympathetic innervation of the head and neck consists of the cervical and upper thoracic sympathetic chain [8]. It is postulated that unrestrained cervical sympathetic activity in the head and neck region can be blocked by injecting local anesthetics in the stellate ganglion, restoring the homeostasis of the regional ANS [9]. The stellate ganglion block (SGB) has been used clinically for medical conditions associated with increased sympathetic nervous system activity [10-12]. In this report, the authors discuss the resolution of persistent anosmia after performing SGB in a patient who had completely recovered from COVID-19 infection, implicating dysautonomia in the pathophysiology of PASC.

This article was previously posted to the QEIOS preprint server on June 11, 2022.

## Case presentation

A 48-year-old female patient, who consented to the publishing of this case report, presented to our clinic four months after recovering from a COVID-19 infection. The patient did not have any significant comorbidities. She reported that she had developed fevers ranging from 99 to 102 °F, non-productive cough, and nasal congestion. The reverse transcriptase polymerase chain reaction test (RT-PCR) performed on the patient’s nasal swab, at a drive-through laboratory, had revealed a positive COVID-19 infection.

The patient was placed on antiviral therapy (a combination of nirmatrelvir and ritonavir tablets known as Paxlovid) for five days, followed by a complete resolution of symptoms. She reported that she had first noticed the loss of smell and taste three to four days into the acute phase of COVID-19 and attributed it to nasal congestion and high fevers. The patient also reported fatigue, light-headedness, and loss of smell and taste. She reported that all her symptoms had resolved within one month, except for issues with taste and smell. She reported a complete loss of sense of smell and altered taste sensation to various types of foods. The patient further reported that she had tried various nasal decongestants and mucolytic agents. She frequently did nasal irrigation with saline. The patient had also failed olfactory threshold tests. No conductive loss of sense of smell was identified, such as nasal obstruction due to rhinosinusitis or allergic rhinitis. The patient also underwent gustatory tests with taste strips and was able to identify only salty and bitter tastes. At six months post-COVID-19 infection, she continued to have anosmia and difficulty tasting sweet and sour foods.

**Figure 1:**
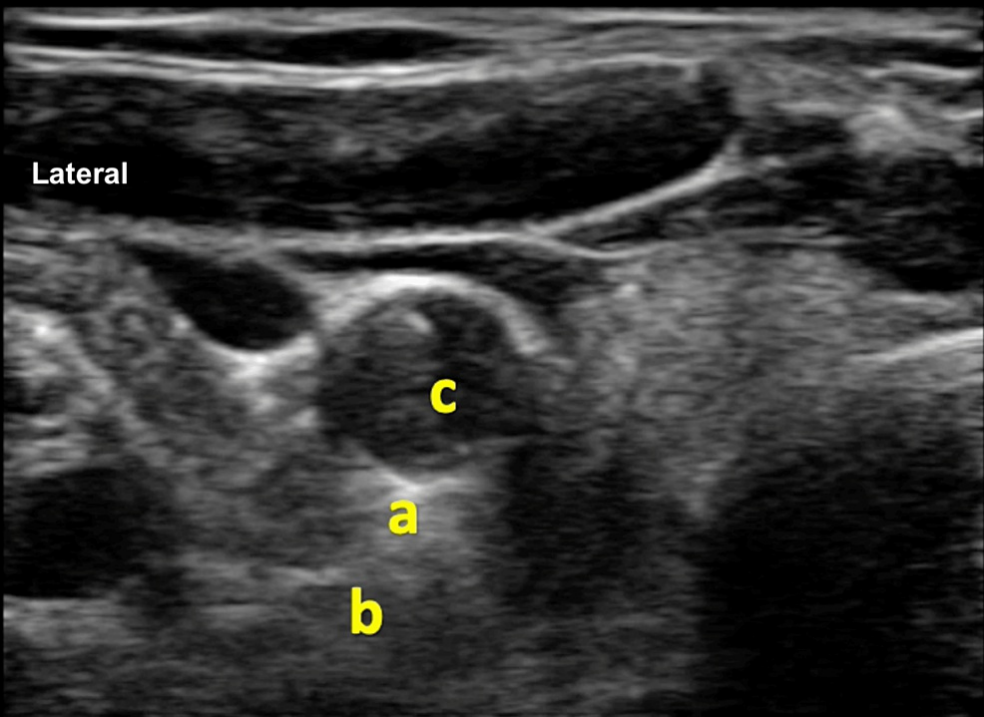
Ultrasonographic image of the right side of the neck depicting stellate ganglion a: stellate ganglion above the longus colli muscle. Site for deposition of local anesthetic solution. b: longus colli muscle. c: right carotid artery

Anosmia and dysgeusia were affecting her quality of life, and she was getting annoyed due to a lack of therapeutic options. She had been finally referred to our clinic by her otolaryngologist for undergoing SGB. The patient was counseled that the authors would perform SGB based on anecdotal examples in the current literature. The patient underwent a right-sided SGB under ultrasonographic guidance, with 4 ml of 0.25% bupivacaine (Figure 1).

The patient reported a partial return of her sense of smell within 24 hours. She then underwent the left-sided SGB after 72 hours and reported a complete resolution of anosmia 24 hours after the second SGB. She reported that her altered taste sensation resolved a few days after the last block.

## Discussion

Anosmia/parosmia as a part of COVID-19 infection could be due to neurological virulence and associated cytopathic effects [13,14]. Regarding patients reporting resolution of anosmia post-SGB, an argument could be made that they might have undergone structural recovery and only need to reset the tone of the ANS to experience functional recovery. Furthermore, a dramatic resolution of anosmia after SGB favors dysautonomia rather than cytopathic or structural damage due to COVID-19 as the underlying etiology.

Dysautonomia is also reported with other viral illnesses (hepatitis C, HIV, Epstein-Barr virus) and other pathologies such as alcoholism, diabetes, and Parkinson's. Dysautonomia is associated with fatigue, anosmia, heart rate variability, bowel and bladder dysfunction, and orthostatic hypotension [6]. Multiple theories have been proposed to explain the persistence of dysautonomia post-COVID-19 infection [15]. Current literature implicates the complex interaction between the angiotensin-converting enzyme 2 (ACE2) receptor and COVID-19 as the underlying mechanism behind dysautonomia and subsequent anosmia. ACE2 enzyme converts angiotensin II (Ang II) to angiotensin (1-7) [16]. Ang inhibits inflammation [17]. ACE2, expressed in membrane-bound and soluble forms, is the receptor for the spike portion of the SARS-CoV-2 virus. A link between ACE2 and the spike portion of SARS-CoV-2 facilitates the viral entry into the cells while inhibiting the activity of the ACE2 enzyme. A subsequent decrease in the activity of ACE2 potentiates inflammation by perpetuating the feed-forward loop mediated by the ATII molecule [18]. Furthermore, auto-antibodies, such as anti-interferon, anti-nuclear, and anti-phospholipids, are ubiquitously detected in patients with PASC. Antibodies to ACE2 enzyme can also perpetuate inflammation and dysautonomia by reducing the activity of both membrane-bound and soluble ACE2 [19-21]. ACE2-viral interaction theory also explains an increased incidence of anosmia in patients of European descent with an increased expression of ACE2 compared to Asians [22]. The expression of ACE2 also increases with age, explaining the less severe prognosis of COVID-19 infection in young adults and children [23]. The female gender, with overexpression of ACE2 as compared to males, has an increased propensity for olfactory issues due to COVID-19 infection [24].

Irrespective of etiology, sequelae of COVID-19 neurotropism and tissue injury entail chronic sympathetic hyperresponsiveness, vasomotor dysfunction, persistent chronic inflammation, and aberrant neuroplasticity manifesting clinically as dysautonomia. Impaired cerebral blood flow (CBF) is a common observation reported in subjects with dysautonomia [6,7,25]. Various reports have established that CBF impairment parallels dysautonomia's clinical severity in patients with PASC [26]. SGB improves CBF under normotensive conditions [27]. The increase in CBF leading to improved perfusion of cortical areas associated with the sense of smell or the peripheral receptors in the facial region might be responsible for the immediate resolution of anosmia seen with SGB. However, the exact mechanism behind the dramatic resolution of anosmia post-SGB is still unknown.

## Conclusions

The mechanistic factors related to the dramatic improvement of anosmia due to SGB are still debatable; however, SGB may be an effective treatment option for patients with olfactory and taste issues associated with PASC. At this point, the evidence for using SGB to alleviate anosmia and dysgeusia associated with Long COVID is anecdotal and limited to a few case reports. Collaborative multi-institutional research might be required to gather more evidence to support using SGB as a treatment modality for anosmia and dysgeusia due to Long COVID.
